# Effect of Pullulan‐Chitosan Film Containing Titanium Dioxide (TiO_2_) Nanoparticle and Tarragon Essential Oil on the Quality Properties During Refrigerated Storage of Rainbow Trout Fillets

**DOI:** 10.1002/fsn3.70717

**Published:** 2025-07-28

**Authors:** Ainaz Khodanazary

**Affiliations:** ^1^ Department of Fisheries, Faculty of Agriculture and Natural Resources Gonbad Kavous University Gonbad Kavous Iran

**Keywords:** biopolymers, nanocomposite film, rainbow trout, tarragon essential oil, TiO_2_ nanoparticle

## Abstract

Nowadays, there is a serious ecological problem with synthetic plastics in seafood packaging because of their total non‐biodegradability. The objective of this study is the estimation of the influence of pullulan (P)‐chitosan (CH) film in combination with titanium dioxide nanoparticles (TiO_2_ NPs) and tarragon essential oil (TEO) on the bacterial counts, physicochemical properties, and sensory analysis of rainbow trout fillets during refrigerated storage. For this aim, the treated fillets were divided into: pullulan‐chitosan (PCH), PCH‐TiO_2_, PCH‐TEO, and PCH/TiO_2_‐TEO. The results demonstrate that the combination of TiO_2_ and TEO with PCH solution is an active nanocomposite film with antimicrobial and antioxidant effects. The wrapped fillets improved the physicochemical properties (such as total volatile bases‐ nitrogen [TVB‐N], pH, peroxide value [POV], and thiobarbituric acid [TBA]) during refrigeration storage. The highest rate of TVB‐N (30.33 mg/N100 g), pH (7.04), POV (1.79 meq peroxide/1000 g lipid), and TBA (3.07 mg MDA/kg) was recorded in PCH/TiO_2_‐TEO wrapped fillets on day 12. The PCH‐TEO was better than the (PCH‐TiO_2_) groups in reducing the rate of oxidation of lipids of rainbow trout fillets. However, there was no significant difference between them in the control of the microbial population. The sensory acceptance score in rainbow trout fillets wrapped with PCH/TiO_2_‐TEO was better than other samples on day 12 (3.66), and it was lower than the critical score for fishery products. Overall, these findings suggest that TiO_2_/TEO PCH‐based nanocomposite film could be utilized as an alternative packaging method in seafood products with notable antioxidant, antibacterial, and nutritional properties.

## Graphical abstract

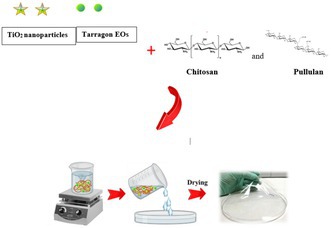

## 1 Introduction

Synthetic plastics have been widely used for their ease of use, cost–benefit, good physical properties, and excellent gas‐barrier properties (Zhao et al. 2020). However, the growing risk of plastic waste has raised concerns about environmental problems because they are non‐degradable (Hadidi et al. 2022). Replacement natural biodegradable polymeric biomaterials, including polysaccharides and proteins, have increasingly attracted the attention of researchers (Vasconez et al. 2009; Dehghani et al. 2017; Shahhoseini et al. 2019). Pullulan (P), as a natural microbial water‐soluble exopolysaccharide, consists of a maltotriose trimer made up of α‐(1 → 6)‐linked (1 → 4)‐α‐d‐triglucosides with a chemical formula of C_6_H_10_O_5_, which is isolated by the culture broth of fungus‐like yeast called *Aureobasidium pullulans* (Singh et al. 2008; Xu et al. 2015). Pullulan has extraordinary film‐forming abilities (Shah Hosseini 2023). The characteristics of the film prepared by pullulan include being colorless, transparent, heat‐sealable, and highly impermeable to both oil and oxygen (Liu et al. 2019; Zhou et al. 2021). Furthermore, pullulan has biocompatibility and biodegradability properties (Ferrari Cervi et al. 2021). Pullulan is an effective and excellent alternative to petroleum‐based polymers. Nevertheless, pullulan has inherent shortcomings such as poor mechanical properties, high hydrophilicity, and lack of active functions (e.g., antioxidant and antibacterial activities), etc., which can increase the properties of pure pullulan films by linking with other polymers through chemical linkages or combining with nanofillers and active compounds into pullulan formulations (Synowiec et al. 2014; Wu et al. 2013; Yan et al. 2020). Biopolymers can supply gas barrier properties for pullulan to produce excellent films (Rahman et al. 2019). Chitosan and pullulan are among the most widely used and commonly investigated biopolymers (Kumar et al. 2021). Chitosan (CH), as a linear polymer of β‐(1‐4)‐linked D‐glucosamine and N‐acetyl‐D‐glucosamine, is obtained by deacetylation of chitin, which is found in the exoskeleton of crustaceans and fungal cell walls (Rivero et al. 2009). CH has some advantages, including edibility, biocompatibility, being nontoxic, barrier properties, and being a carrier of food additives such as antioxidant and antimicrobial agents. Chitosan with a positively charged surface (NH^3+^) can interact electrostatically with pullulan with negatively charged pullulan (OH^−^), leading to the formation of pullulan‐CH complexes. Li et al. (2015) and Wu et al. (2013) indicated that pullulan‐chitosan could improve their barrier and mechanical properties, as well as antimicrobial and antioxidant properties.

In recent years, metallic nanoparticles such as titanium dioxide nanoparticles (TiO_2_ NPs), also called titania, interact with the film matrix as novel antimicrobial nanofillers to increase both physical and antimicrobial functions of films (Roy et al. 2010; Shankar and Rhim 2015). Also, nanoparticles can control the degradation process of polymers (Goñi‐Ciaurriz and Vélaz 2022). According to the Food and Drug Administration (FDA), TiO_2_ nanoparticles (TiO_2_ NPs) are safe as a food additive, and the amount of TiO_2_ NPs added to the food should not be more than 1% of the total mass of the food (CFR n.d.). TiO_2_ NPs (20–400 nm) have some excellent characteristics, including economical, abundant element, low toxicity, antibacterial activity, and biocompatibility, that make them suitable for the fabrication of natural nanocomposite films (Hou et al. 2019; Zhang and Rhim 2022). In the matter of antioxidant properties, Taheri et al. (2018) and Alizadeh‐Sani et al. (2020) stated that TiO_2_ NPs are found to affect lipid oxidation in the food samples due to their free radical scavenging property. There is much research that combines polymers and TiO_2_ NPs in order to delay the growth of bacteria (Hosseinzadeh et al. 2020; Sayadi et al. 2022). In addition, natural bioactive compounds like EOs and extracts are used to enhance the antioxidant and antibacterial activities of the edible films (Safari et al. 2018; Javadian et al. 2017; Bagheri et al. 2016). Tarragon essential oil (TEO) ( *Artemisia dracunculus* ) has valuable biological effects such as antioxidant and antimicrobial activities (Liu et al. 2018). Flavonoids, phenolic acids, and their esters are some common sources of polyphenols of TEO. EOs cannot be used directly because of the vulnerability of their properties due to interactions between volatile oxidation compounds and environmental agents (Sharma et al. 2020). The application of biopolymers and EOs was widely studied to prolong the quality of fish fillets (Sharma et al. 2020; Kakaei and Shahbazi 2016; Alp‐Erbay et al. 2017). Matrix polymers can combine with TiO_2_ and EOs to improve the mechanical characteristics, water vapor permeability, and antibacterial and antioxidant activities (Alizadeh‐Sani et al. 2017; Gohargani et al. 2020; Yang et al. 2022). Eshaghi et al. (2024) stated that active films containing TiO_2_ NPs and EOs prolonged the shelf life of common carp fillet by controlling microbial growth and the formation of free radicals. The application of biopolymers incorporating nanoparticles and essential oils is a necessary component in the novel and active packaging industry. As far as we know, no edible film has been developed using a combination of pullulan/chitosan, TEO, and TiO_2_ NPs. Therefore, the goal of the present research is to study the effect of pullulan‐chitosan composite film containing TiO_2_ and TEO on the physicochemical, microbiological, and sensory properties of rainbow trout fillet.

## 2 Materials and Methods

### 2.1 Materials

Pullulan was purchased from Shandong Freda Biotechnology Co. Ltd., China. Chitosan with medium molecular weight (≥ 92.0% deacetylation) was prepared from Sigma Chemical Company. TiO_2_ powder (mean diameter, about 30 nm) was purchased from Iranian Nanomaterials Pioneers Company, Mashhad, Iran. Tarragon essential oil was prepared from Hirad Commercial Company (Iran).

### 2.2 Preparation and Treatment of Fish Samples

The average weight of rainbow trout bought at a local market was 520–635 g and they were transferred to the Seafood Processing Laboratory. Two fillets were prepared by removing the head and cutting through the belly side of the fish. Film‐forming solutions were prepared by dissolving pullulan and chitosan separately. Pullulan and chitosan solutions were prepared separately. Chitosan solution was prepared by dissolving 1% (w/v, film‐forming solution) of chitosan in an aqueous solution of glacial acetic acid (1% v/v, film‐forming solution) and stirring on a magnetic stirrer plate for 2 h (Ojagh et al. 2010). At the same time, pullulan was completely dissolved in 2.5% w/w sterile deionized water (Kamali et al. 2022). Afterwards, the two solutions were mixed in a 1:1 ratio. To this solution, TiO_2_ NP 10% (w/w based on PCH) (Khodanazary 2024) and TEO 2% (w/w based on PCH) (Farsanipour et al. 2020) were added and placed on a magnetic stirrer at room temperature for 15 min. Glycerol (1.0%, v/v, film‐forming solution) was dissolved in the above solution as a plasticizer. After stirring for 1 h, the solution was deposited into a 90 mm Petri dish. The composite solution was removed of bubbles by vacuumizing and spread evenly in a Teflon dish. It was dried at 30°C with 50% relative humidity for 24 h to obtain the films. Lastly, the films were peeled off from the casting plate. The fillet samples were randomly divided into four groups and in triplicate (in 12 packages). The treatments included the (1) PCH, (2) PCH‐TiO_2_, (3) PCH‐TEO, and (4) PCH‐TiO_2_‐TEO. Fillets were wrapped with these films and sealed in polyethylene bags (3 samples for each treatment), maintained at 4°C for 12 days to evaluate quality properties at 5 different days (0, 3, 6, 9, and 12 days).

### 2.3 Film Tests

#### 2.3.1 Physical Properties

*Thickness measurement*: A micrometer with an accuracy of 0.01 mm was used to determine the thickness of the films. Measurements were made at 3 different points in the film, and then averaged. The calculated average thickness was used to determine water vapor permeability. *Moisture measurement*: Moisture content of films was determined by measuring the weight loss of films upon drying in an oven at 110°C until a constant weight was reached (dry sample weight). *Water vapor permeability coefficient*: The WVP of the film samples was measured gravimetrically according to the ASTM E96‐05 standard method. In this regard, glass vials used for this experiment were filled with dried anhydrous CaSO_4_ to reach the vials inside RH to 0% and sealed with film samples, and then each vial was placed in a desiccator containing K_2_SO_4_ solution to maintain the RH of 97% at 25°C. The vials were weighed periodically every 24 h using a digital balance with an accuracy of ±0.0001 g. Consequently, the slope (changes of weight vs. time) was calculated by a linear regression.

#### 2.3.2 Measurement of Antioxidant Properties

The antioxidant activity of the films was investigated using the DPPH free radical scavenging method (Aayush et al. 2024). Pieces of film (6 cm^2^) were immersed in methanol and subjected to vigorous stirring and vortexing for 10 min to digest the film and release antioxidant compounds. After centrifugation for 10 min at 3000 rpm, the methanol supernatant was used to determine the inhibitory power of DPPH. 2 mL of DPPH methanolic solution (0.06 mM) was mixed with 1 mL of sample supernatant and 1 mL of methanol. The control sample was prepared by adding 2 mL of methanol in the absence of film solution. After stirring for 1 min, the mixture was placed in a dark place at 25°C for 30 min, and then its adsorption was measured at 517 nm by spectrophotometer (TOG/TPH, Dr‐4000).

#### 2.3.3 Measurement of Antibacterial Properties

For this purpose, culture medium of *Escherichia coli* ATCC 25922 and *Staphylococcus aureus* ATCC 33591 was prepared. The produced film was then cut into a circle with a diameter of 9 mm (He et al. 2021). Nutrient agar medium was used for the culture of *Escherichia coli* and *Staphylococcus aureus* . After culturing the bacteria, the cut film pieces were placed on the culture surface. After 48 h of storage at 37°C, the amount of halos created around the film was measured 3 times from different points and their average was recorded.

### 2.4 Measurement of Quality Properties of Wrapped Fillets

#### 2.4.1 Bacteriological Analyses

Total mesophilic bacteria and psychrophilic bacteria were enumerated using the petri surface smear method (ICMSF 1986). These petri dishes were incubated at 30°C for 48 h for mesophilic counts and at 5°C for 10 days for psychrophilic viable counts. One milliliter of dilution was employed for bacterial culturing. Plate agar counting (PCA) was used to determine total mesophilic bacteria (TMB) and total psychrotrophic bacteria (TSB) of fish fillet through the spreading plate approach following the incubation of plates for 2 days at 35°C and for 7 days at 4°C, respectively.

#### 2.4.2 Physicochemical Analysis

Total volatile basic nitrogen (TVB‐N) value was estimated by the micro‐diffusion method (Goulas and Kontominas 2005). The micro‐diffusion method was determined by distillation after the addition of MgO to the homogenized fish sample. The distillate was collected in a flask containing an aqueous solution of boric acid and methyl red as an indicator. Afterward, the boric acid solution was titrated with sulfuric acid (H_2_SO_4_) solution. The TVB‐N value (mg N/100 g of fish) was determined according to the consumption of sulfuric acid. A pH meter was employed to determine the pH of fish fillets. The peroxide value (POV) of the fillets was measured using Woyewoda's method (Woyewoda et al. 1984). Results were expressed in meq peroxide/1000 g lipid. Thiobarbituric acid (TBA) measurement was determined following Siripatrawan and Noipha (2012) with some modification. Ten grams of homogenized sample were added with 97.5 mL of distilled water and 2.5 mL of 4 M HCl. The mixture was heated with steam distillation. Five milliliter of distillate was added to 5 mL of thiobarbituric reactive reagent containing 0.02 M TBA in 90% glacial acetic acid and incubated in boiling water for 35 min. After cooling, the absorbance of the pink solution was measured at 532 nm using a spectrophotometer. The TBA value is expressed as mg malonaldehyde equivalents/kg sample.

### 2.5 Sensory Analysis

Sensory evaluation was determined with 32 trained members (15 males and 17 females, aged 26 to 33 years) previously trained according to ISO 8586. For sensory analysis, a quantitative descriptive analysis was used. The 9‐point hedonic scale was applied using some attributes including color, odor, and overall preference (1‐dislike extremely, 2‐dislike very much, 3‐dislike, 4‐dislike slightly, 5‐neither like nor dislike, 6‐like slightly, 7‐like, 8‐like very much and 9‐like extremely) (Ehsani et al. 2016). The color and odor of fresh fish fillets were dark and bright red, and fresh or neutral odor (score = 9), respectively. On the other hand, the pale red or brownish/discolored and putrid odor (score = 1) indicated spoiled fish.

### 2.6 Statistical Analysis

The one‐sample K–S test was employed to determine the normal distribution of data. All measurements were replicated three times for each lot, and a completely randomized design was used. Mean values ± standard error were reported for each case. Significant differences between factors and levels were determined by one‐way analysis of variance (ANOVA) using SPSS 16.0 for Windows (SPSS Inc., Chicago, Illinois, USA). Moreover, Duncan's multiple range test was performed to detect the significant difference. A level of *p* < 0.05 was considered significant. The Friedman test was performed for analyses of the non‐parametric data (sensory analysis).

## 3 Results and Discussion

### 3.1 Physical Properties of Films

The effect of incorporating TiO_2_ and TEO on the physical properties of PCH‐based film is summarized in Table 1. The thickness of the films ranged between 0.036 and 0.087 mm. With the incorporation of TiO_2_ or TEO into PCH, the moisture content decreased, which is attributed to the compactness of the film network. The TiO_2_ or TEO might cause a decrease in the availability of hydroxyl and amino groups and limit polysaccharide–water interactions by hydrogen bonding, resulting in a decrease in the moisture content value of edible films because of the formation of covalent bonds between the functional groups of the chitosan chain (Flórez et al. 2022). Water vapor permeability coefficient (WVPC) is a main factor in food packaging biopolymers because water is an important factor in food spoilage reactions. The WVPC value is measured at 2.08 g s^−1^ m^−1^ Pa s^−1^ × 10^−10^ in PCH film. The WVPC of PCH films was slightly enhanced by mixing with TEO (*p <* 0.05). The same results were obtained by Qin et al. (2024). When TEO and TiO_2_ were incorporated into the PCH film formulation, the WVPC of the film decreased due to the hydrophobic nature of TEO and the particle size of TiO_2_ in the films, which could affect the hydrophilic/hydrophobic property of the films with TEO and create complex pathways for water to pass through the polymer matrix with TiO_2_ (Ojagh et al. 2010).

**TABLE 1:** Physical properties of PCH films incorporated with TiO_2_ and TEO.

Physical properties	Treatments	
Thickness (mm)	PCH	0.084 ± 0.00^a^
	PCH‐TiO_2_	0.087 ± 0.00^a^
	PCH‐TEO	0.036 ± 0.01^a^
	PCH‐TiO_2_‐TEO	0.086 ± 0.00^a^
Moisture (%)	PCH	23.56 ± 1.20^a^
	PCH‐TiO_2_	18.22 ± 0.57^b^
	PCH‐TEO	13.44 ± 0.40^c^
	PCH‐TiO_2_‐TEO	12.26 ± 0.16^c^
Water vapor permeability coefficient (g s^−1^ m^−1^ Pa s^−1^ × 10^−10^)	PCH	3.13 ± 0.06^a^
	PCH‐TiO_2_	2.28 ± 0.08^b^
	PCH‐TEO	2.04 ± 0.01^b^
	PCH‐TiO_2_‐TEO	1.68 ± 0.12^c^

*Note:* Means in each column with different superscript letters are significantly different (*p* < 0.05).

### 3.2 Antioxidant Activity of Films

Antioxidant properties of additive compounds promote extending the food shelf life to improve packaging. The demand for antioxidant active food packaging is increasing due to its unquestionable advantages compared with the direct addition of antioxidants in food samples (Dairi et al. 2019). In this way, the antioxidant activities of the PCH, PCH‐TiO_2_, PCH‐TEO, and PCH‐TiO_2_‐TEO formulation films were evaluated by the DPPH radical scavenging ability. The pure PCH film demonstrated low antioxidant ability under the time‐dependent condition (Table 2). This demonstrated that the pure CH film showed weak antioxidant activity in the PCH‐based film. The addition of TEO or TiO_2_ significantly increased the antioxidant activity of the CH film because of the presence of polyphenols in TEO, the functional groups on the surface of TiO_2_ nanoparticles, the nano‐size of TiO_2_, and its higher surface area. Aljabeili et al. (2018) reported that films containing TEO have higher radical scavenging potency because of its main phenolic compounds such as thymol, 1,8‐Cineole, and γ‐Terpinene. Liu et al. (2021) and Sarıcaoglu and Turhan (2020) believed that the antioxidant activity of the films enriched with TEO was mainly derived from thymol. As observed, the PCH‐TEO samples had higher antioxidant ability than the corresponding PCH‐TiO_2_ samples. On the other hand, the DPPH content of PCH‐TEO and PCH‐TiO_2_‐TEO was the same. Moreover, the mechanism of antioxidant could be correlated with the efficiency of the metal ion chelating effect. Similarly, Almasi et al. (2020) and Sun et al. (2020) observed higher antioxidant activity in biopolymer films loaded with EOs due to the phenolic and terpenoids of EOs.

**TABLE 2:** Antioxidant and antimicrobial activities of the PCH based film containing TiO_2_ and TEO.

Film	DPPH (%)	Non growth halo (mm^2^)	
		*E. coli*	*S. aureus*
PCH	20.67 ± 29^c^	2.00 ± 0.57^d^	2.66 ± 0.33^d^
PCH‐TiO_2_	63.27 ± 0.95	6.33 ± 0.33^b^	7.33 ± 0.33^b^
PCH‐TEO	82.90 ± 2.19^a^	4.66 ± 0.33^c^	6.00 ± 0.00^c^
PCH‐TiO_2_‐TEO	83.24 ± 1.51^a^	7.66 ± 0.33^a^	8.33 ± 0.33^a^

*Note:* Means in each column with different superscript letters are significantly different (*p* < 0.0).

### 3.3 Antibacterial Activity of Films

The antibacterial activity of PCH, PCH‐TiO_2_, PCH‐TEO, and PCH‐TiO_2_‐TEO formulation films was evaluated by the inhibition zone test by agar diffusion disc against bacteria. *E. coli* and *S. aureus* were selected as representative Gram‐negative and Gram‐positive bacteria for the antibacterial test in food contamination. The PCH exhibited weaker antibacterial activity than the PCH‐TEO and PCH‐TiO_2_ samples. Conversely, PCH‐TiO_2_, PCH‐TEO, and PCH‐TiO_2_‐TEO indicated strong antibacterial activity with a clear inhibitory zone by the absence of bacterial growth around the film cuts. The diameter of the bacteriostatic zone of the composite film solution containing TiO_2_ was larger than that of the PCH‐TEO film solution (Table 2). The PCH‐TiO_2_‐TEO exhibited a higher antibacterial activity than the PCH‐TiO_2_ and PCH‐TEO, indicating that TiO_2_ could play an additive antibacterial role with TEO. The additive interaction between PCH and TEO decreased the loss of EOs during processing, leading to active nanocomposites with improved antimicrobial properties. Mechanisms of antibacterial activity of TiO_2_ are still under survey. The antibacterial activity of TiO_2_ NPs film could relate to Ti^4+^ ions as a vital for antimicrobial activities. The antibacterial mechanism was speculated to be that TiO_2_ NPs could disrupt the cell membrane with interaction with sulfur‐containing proteins and phosphorus‐containing compounds like DNA, causing cell lysis, and also the disruption of the peptidoglycan wall and cytoplasmic membranes through electrostatic attractions between positively charged nanoparticles and negatively charged bacterial cells (Serov et al. 2024). The hydrophilic outer membrane of Gram‐negative bacteria acts as a molecular filter for hydrophilic compounds and hinders their permeability. By adding TEO to PCH‐TiO_2_, the antibacterial activity of the films on *E. coli* and *S. aureus* increased from 9.44 to 12.37 mm. Some researchers stated that the composite films enriched with TEO possessed a greater inhibitory impact on *S. aureus* than *E. coli* (Sarıcaoglu and Turhan 2020; Qin et al. 2024). EO could compound with polysaccharides, phospholipids, and fatty acids, leading to destruction and penetration of the lipid structure of the bacterial cell membrane (Qin et al. 2024).

### 3.4 Bacteriological Analysis

**FIGURE 1:**
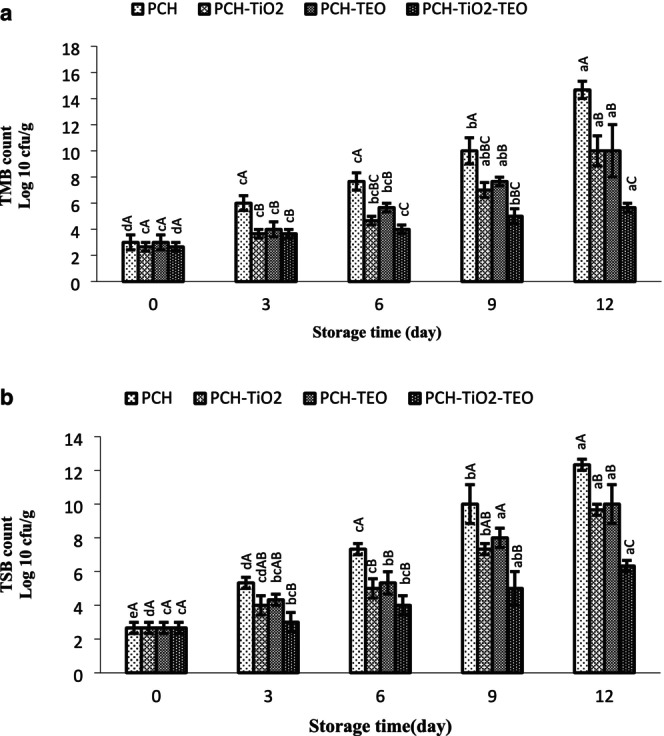
(a, b) Changes in TMB and TSB counts of rainbow trout fillets during refrigerated storage. Mean values and standard errors from the three replicates are presented. The different capital letters within the same storage time indicate the significant differences (*p* < 0.05). The different small letters within the same treatment indicate the significant differences (*p* < 0.05).

Protein is one of the most abundant compounds in seafood, and microorganisms and enzymatic action cause protein decomposition in seafood products (Singh et al. 2016). The microbiological quality of treated fish fillets is depicted in Figure 1a,b. Among the psychrotrophilic bacteria, the Gram‐negative bacteria, including *Acinetobacter*, *Pseudomonas*, and *Shewanella*, are the predominant genera of bacteria responsible for the spoilage of cold‐stored rainbow trout (Du et al. 2022). The initial total mesophilic bacteria (TMB) (log_10_ CFU/g) in the PCH was 3.00 log_10_ CFU/g; it was 2.66, 3.00, and 2.66 log_10_ CFU/g for PCH‐TiO_2_, PCH‐TEO, and PCH‐TiO_2_‐TEO, respectively, showing the acceptable bacterial quality of the fish fillets (Karim 2013). The rainbow trout fillet's initial total psychrotrophic bacteria (TSB) count was 2.66 log_10_ CFU/g. The result of bacteria at day 0 agrees with that reported by Nowzari et al. (2013). The TMB count increased (*p* < 0.05) during storage in the refrigerator for 12 days and reached 14.66, 10.00, 10.00, and 5.66 log_10_ CFU/g for PCH, PCH‐TiO_2_, PCH‐TEO, and PCH‐TiO_2_‐TEO, respectively, at the end of storage. Also, the TSB counts were significantly increased (*p* < 0.05) and reached 12.33, 9.66, 10.00, and 6.33 log_10_ CFU/g for PCH, PCH‐TiO_2_, PCH‐TEO, and PCH‐TiO_2_‐TEO, respectively, at the end of storage. The increasing rate of TMB and TSB counts in treated samples is slower than in PCH, indicating that the wrapped samples with the PCH film had minimal effect on decreasing the bacterial growth trend because of the semipermeable film of PCH. Besides, chitosan has antibacterial properties due to (1) interaction of the NH_3_^+^ group of glucosamine monomer in chitosan molecules with negative charges of macromolecules on the bacteria cell surface; (2) chelating metal ions with unprotonated amino groups of chitosan on the cell surface to disrupt cell walls or membranes; (3) the binding of chitosan with DNA to prevent mRNA synthesis and protein of the microorganisms (Goy et al. 2016). Among all of the treatments, the samples wrapped with PCH‐TiO_2_‐EOs had the lowest abundance of TMB and TSB (*p* < 0.05). While PCH had a low effect on decreasing viable bacterial growth, TiO_2_ nanoparticles and TEO could increase the antibacterial properties. PCH‐TiO_2_ exhibited higher antibacterial activity than PCH‐TEO, indicating that the antibacterial activity of TiO_2_ was better than that of TiO2, and the presence of TEO and TiO_2_ together within the nanocomposite film had an additive effect, leading to the most significant reduction in the bacteria counts. Similar results were reported by Sayadi et al. (2022). Remarkably, the inclusion of TiO_2_ and TEO in the PCH solution strengthened its antibacterial activity toward the entire observed bacterial groups. Therefore, PCH‐TiO_2_‐TEO had the best antibacterial activity among the four composite films. The antibacterial mechanism of TiO_2_ may be due to (1) its photocatalytic properties: the generation of hydroxyl radicals and reactive oxygen species (ROS) destroys the outer membrane of the bacteria by oxidizing the polyunsaturated phospholipid components of the cell membrane of the bacteria and reacts with the key cellular enzymes as well; (2) the TiO_2_ nanoparticles and the bacterial cells have opposite charges: the electrostatic attraction between bacterial cells and TiO_2_ nanoparticles results in disturbance of the cell membrane of the bacteria (Alizadeh‐Sani et al. 2020; Feris et al. 2009; Planchon et al. 2013; Shibata 2010). Several studies have shown that TiO_2_ NPs have antimicrobial activities (Othman et al. 2014; Yuan et al. 2023). Sharma et al. (2023) confirmed the antibacterial properties of TiO_2_ NP against *E. coli* and *S. aureus* in the PLA/PBAT composite blend films. In another study, Xu et al. (2017) found that the graphene oxide/chitosan biopolymer/TiO_2_ films limit the growth of *Aspergillus niger* and *Bacillus subtilis* Khashan et al. (2021) reported that the antibacterial activity of TiO_2_ NPs reduced microbial growth ( *E. coli* , *Pseudomonas aeruginosa* , *Proteus vulgaris* , and *S. aureus* ). Contrary to previous studies, Lin et al. (2015) observed that chitosan‐TiO_2_ had no inhibitory effect on the culture medium. According to Esfahani et al. (2020), sago starch reinforced with cinnamon essential oil and TiO_2_ NPs has antimicrobial activity against microorganisms ( *Salmonella typhimurium* , *E. coli* , and *S. aureus* ). Generally, Gram‐positive bacteria appear to be more susceptible to metal oxides‐NP than Gram‐negative bacteria due to structural and polarity differences in the bacterial cell membrane (Espitia et al. 2012; Akbar and Anal 2014). Esfahani et al. (2020) revealed that Gram‐positive bacteria were more susceptible than Gram‐negative ones. Adding TEO to the PCH/TiO_2_ solution could be due to the additive effect of the antimicrobials in these samples.

Essential oils contain bioactive compounds including phenolic acids and terpenoids, etc., leading to strong antimicrobial properties in food products (Baptista et al. 2020). The antimicrobial activity of TEO has been ascribed to the presence of sabinene, β‐terpinene, terpinene‐4‐ol, and α‐pinene (Liu et al. 2018). These compounds can influence cell metabolism, causing the death of bacterial cells due to: (1) hydrolyzing the phospholipid bilayer of the bacterial membrane (Burt 2004); (2) interactions of polyphenols with cell envelope transport proteins and nonspecific interactions with carbohydrates (Cowan 1999); (3) the inhibition of bacterial nucleic acid synthesis (Rodríguez et al. 2016). Esmaeili and Khodanazary (2021) found that adding TEO to chitosan solution caused the additive effect of the antibacterial activity. Alizadeh Behbahani et al. (2017) and Raeisi et al. (2012) found tarragon essential oil has an inhibitory effect against the spoilage bacteria of various foods. The recommended content for TMB of fresh fish is 7 log CFU/g (ICMSF 1986). By day 6 of storage, TMB in rainbow trout fillet reached about 7 log_10_ CFU/g (7.66 log_10_ CFU/g) for PCH samples, which is higher than the maximal permissible levels in raw fish, showing a microbiological shelf life of about 5–6 days for the control samples. Samples wrapped with PCH‐TiO_2_ and PCH‐TEO films were suitable for eating for up to 9 days of storage. TMB counts for PCH‐TiO_2_‐TEO films did not exceed the allowable limits at the end of the storage period.

### 3.5 Physicochemical Analysis

**FIGURE 2:**
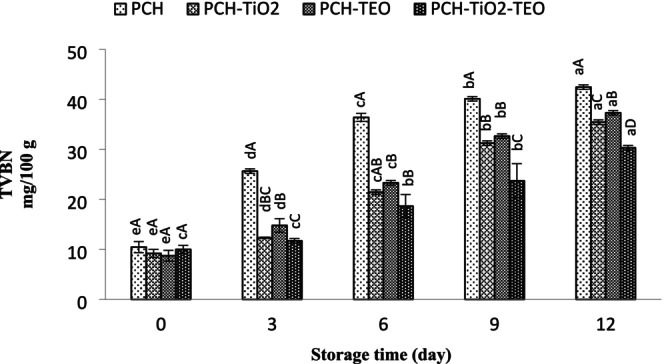
Changes in TVBN value of rainbow trout fillets during refrigerated storage. Mean values and standard errors from the three replicates are presented. The different capital letters within the same storage time indicate the significant differences (*p* < 0.05). The different small letters within the same treatment indicate the significant differences (*p* < 0.05).

The TVB‐N test, including ammonia, primary, secondary, and tertiary amines, is measured as a spoilage index to evaluate raw fish's quality (Benjakul et al. 2003). Figure 2 presents the changes in TVB‐N contents of all samples during the entire storage time. At day 0, the TVB‐N values in treated samples ranged between 8.73 and 10.46 mg N/100 g of flesh, indicating the acceptable quality of raw fish. The TVB‐N values of the treated samples increased significantly with prolonging the storage time (*p* < 0.05). The highest TVB‐N value was observed in the PCH sample, followed by fillet wrapped with PCH‐TiO_2_‐, PCH‐TEO‐, and PCH‐TiO_2_‐TEO, respectively. The increase in TVB‐N value can be explained as follows: (1) the bacterial enzymes produce nitrogenous compounds; and (2) the activity of the endogenous enzymes of the muscle (Alizadeh‐Sani et al. 2020). Therefore, the higher value of TVB‐N is an index of the fillet spoilage. TVB‐N values of rainbow trout treated with TiO_2_ were lower than fish treated with TEO, suggesting TiO_2_ NPs are most effective in decreasing the degradation of nitrogenous compounds of rainbow trout fillets. In comparison, the PCH‐TiO_2_‐TEO film showed the highest microbial properties during the storage, resulting in a lower effect of bacterial enzymes, thereby lowering the TVB‐N value of these samples. Similar results for seafood fillets wrapped/or coated with EOs/and metal nanoparticles incorporated in biopolymer film/or coating were reported (Mosavinia et al. 2022). Sayadi et al. (2022) reported that a decrease in TVB‐N values in samples treated with alginate‐based film incorporated with cumin essential oil and TiO_2_ nanoparticles could prolong the shelf life of beef meat during storage. It can be inferred that PCH film in combination with TiO_2_ NPs and TEO as antimicrobial materials is more effective than other films in controlling the TVB‐N content of fillets, suggesting that PCH film demonstrated a synergistic effect in retarding the TVB‐N value when used in combination with TiO_2_ NPs and TEO. A level of 20–30 mg N/100 g is the acceptable level of TVB‐N in seafood. Therefore, the best storage period for rainbow trout fillets treated with PCH was on day 6, while PCH‐TiO_2_ NPs wrapped fillets or PCH‐TEO wrapped fillets reached this limit on days 9 and 12 of the storage period, respectively, indicating delayed microbial and chemical spoilage.

**FIGURE 3:**
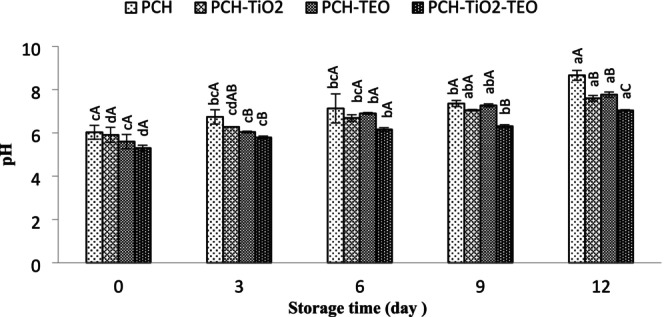
Changes in pH of rainbow trout fillets during refrigerated storage. Mean values and standard errors from the three replicates are presented. The different capital letters within the same storage time indicate the significant differences (*p* < 0.05). The different small letters within the same treatment indicate the significant differences (*p* < 0.05).

The efficiency of the treated groups on pH contents during storage at refrigerator is shown in Figure 3. The initial pH content was 5.37 ± 0.11, which was within the normal pH content range (Arfat et al. 2015). After the fish death, the conversion of glycogen to lactic acid leads to the decrease in pH content. The pH value significantly increased (*p* < 0.05) to 7.43 in the PCH‐, 6.36 in the PCH‐TiO_2_‐, 6.22 in the PCH‐TEO‐, and 6.22 in the PCH‐TiO_2_‐TEO‐treated samples after 12 days of storage. This enhancement is related to (1) the bacteriological degradation of protein by producing alkaline substances such as ammonia, trimethylamine, and volatile basic compounds (Nirmal and Benjakul 2011); (2) the decomposition of fish protein under sufficient oxygen (Esmaeili and Khodanazary 2021). Based on our findings, the pH content increase rate in the PCH was considerably higher than in the other treated samples. The PCH samples indicated higher pH content compared to other samples. It can be attributed to bacterial high growth in these samples, exhibited in the bacterial section results. Among all of the treatments, PCH‐TiO_2_‐TEO‐treated samples had the lowest pH value.

Therefore, retardation in the increase of the pH content in nanocomposite films containing TiO_2_ and TEO could be attributed to the additive antimicrobial effects of these additives against bacterial growth and to inhibit the decomposition of proteins and other nitrogenous compounds (Alizadeh‐Sani et al. 2020). Similar observations were found by Alizadeh‐Sani et al. (2020) for lamb meats treated with whey protein and cellulose nanofiber matrix films incorporating rosemary oil–TiO_2_, and also Sayadi et al. (2022) for beef meat treated with nanocomposite alginate‐based film containing cumin essential oil‐TiO_2_. Although, Bahram et al. (2016) indicated that incorporating cinnamon essential oil in whey protein concentrated coating had no significant effect on pH content.

**FIGURE 4:**
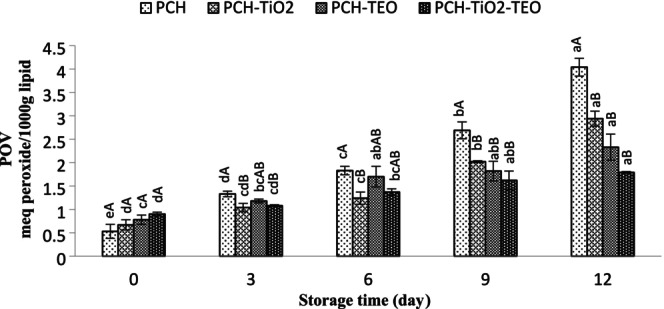
Changes in POV value of rainbow trout fillets during refrigerated storage. Mean values and standard errors from the three replicates are presented. The different capital letters within the same storage time indicate the significant differences (*p* < 0.05). The different small letters within the same treatment indicate the significant differences (*p* < 0.05).

**FIGURE 5:**
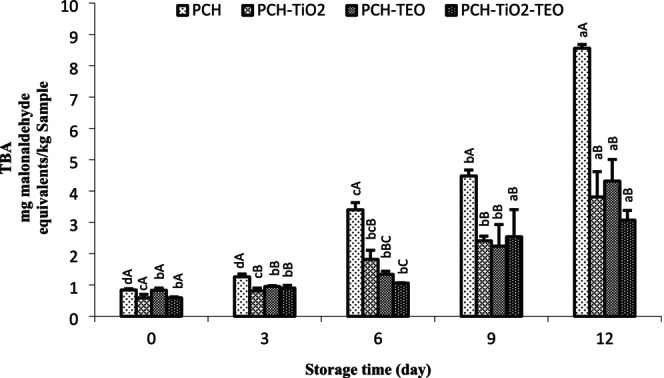
Changes in TBA value of rainbow trout fillets during refrigerated storage. Mean values and standard errors from the three replicates are presented. The different capital letters within the same storage time indicate the significant differences (*p* < 0.05). The different small letters within the same treatment indicate the significant differences (*p* < 0.05).

Variations in the POV of rainbow trout fillets during storage at refrigerator are shown in Figure 4. At day 0, the POV content ranged from 0.04 to 0.06 meq peroxide/1000 g lipid. A gradual increase in POV content in all groups was observed (*p* < 0.05), but the PCH‐TiO_2_‐TEO wrapped fillet slowed the production of POV during storage. The highest POV content was for the 12th day of control samples (1.94 meq peroxide/1000 g lipid). All of the edible films showed less POV than PCH, which means that TiO_2_ NPs and TEO could reduce the lipid oxidation of fillets. The TBA test, as an essential index, measures malondialdehyde (MDA) (the secondary products of unsaturated fatty acids) (Heydari‐Majd et al. 2019). The increase in TBA content indicates lipid rancidity of seafood, which leads to the production of an unpleasant smell and taste and shortens shelf life (Alsaggaf et al. 2017). The TBA content of all the samples is stated in Figure 5. The initial TBA values of all samples were 0.59–0.84 mg MDA/kg, indicating the freshness of the fillet. These results are in agreement with the results found by Nowzari et al. (2013). The TBA value of the treated groups remarkably increased (*p* < 0.05). The fluctuation of TBA contents of all the groups was observed during the storage period. TBA content for PCH‐, PCH‐TiO_2_‐, PCH‐TEO‐, and PCH‐TiO_2_‐TEO‐treated fillet was 8.56, 3.81, 4.32, and 3.07 mg MDA/kg sample, respectively, on day 12. According to the result of TBA and POV values, it is demonstrated the insignificant inhibitory activity of PCH film. PCH‐TEO exhibited the highest antioxidant activity. On the other hand, the antioxidant activity of PCH‐TiO_2_ was better than that of the PCH‐TiO_2_‐TEO. It should be noted that the antioxidant effect of PCH and PCH‐TiO_2_ was the same. These results suggest that interactions among PCH, TiO_2_, and TEO to immobilize TEO and prevent free interaction with the oxidizing free radicals could lead to reduced antioxidant activity of the PCH‐TiO_2_‐TEO (Riahi et al. 2021). The presence of some antioxidant peptides in chitosan plays as antioxidant agents in PCH films. Lan et al. (2021) believed the nanocomposite containing TiO_2_ NP had a strong inhibitory effect on the DPPH radical scavenging activity. However, several researchers showed that the incorporation of different nanoparticles or essential oils in chitosan coating or film improved the inhibition of oxidation (Vieira et al. 2019; El‐Obeid et al. 2018; Duan et al. 2010; Heydari‐Majd et al. 2019). Feng et al. (2019) reported that whey protein isolated edible coatings incorporated with TiO_2_ failed to inhibit lipid oxidation significantly. According to the maximum acceptable levels of TBA of the seafood (1–2 mg MDA/kg) (Lakshmanan 2000), the PCH, PCH‐TiO_2_, PCH‐TEO, and PCH‐TiO_2_‐TEO samples were spoiled on days 6, 9, 9, and 9, respectively. Our results suggest that PCH incorporated with TiO2 and TEO could preserve rainbow trout fillets from the oxidation of lipids.

### 3.6 Sensory Analysis

**FIGURE 6:**
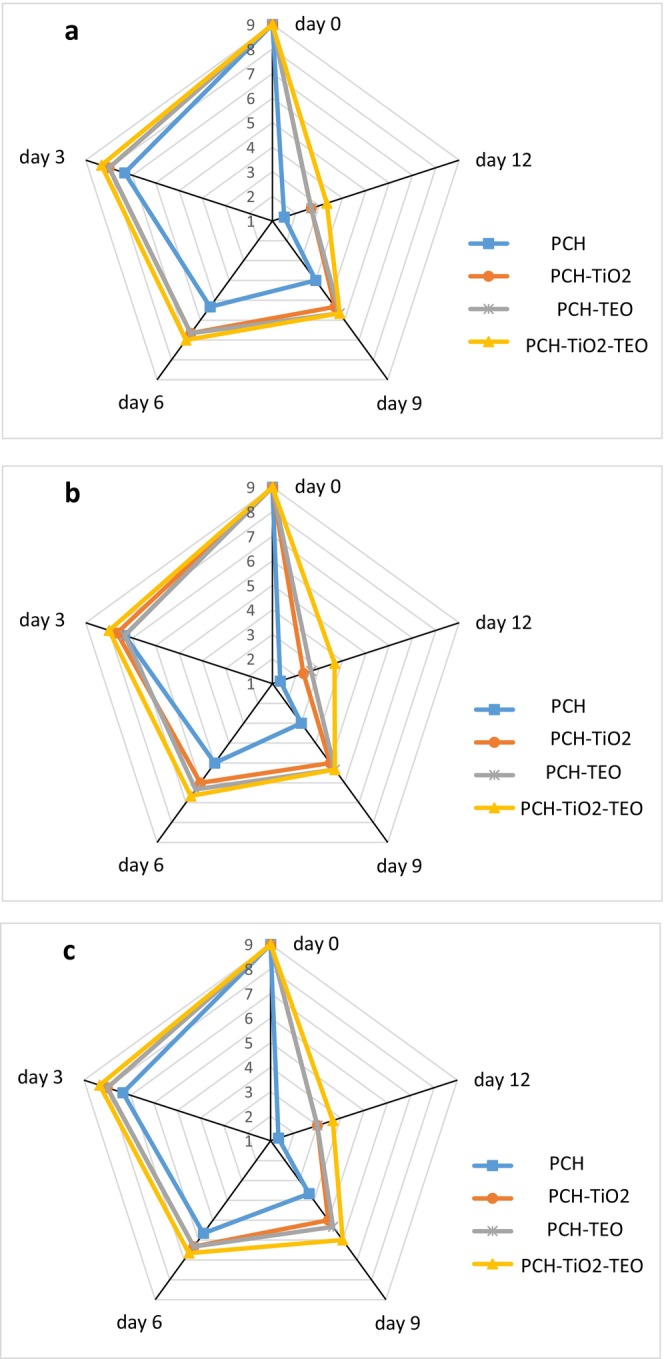
Changes in (a) color; (b) odor; (c) overall preference of rainbow trout during storage at refrigerator.

Sensory attributes (color, odor, and overall acceptability) scores of all treatments reduced as the time of storage increased (Figure 6). The 9‐point hedonic scale was applied for measuring sensory properties: 9 being the highest quality and higher scores (0) indicating worse quality (Ehsani et al. 2016). The sensory properties had decreased with increasing storage time (*p* < 0.05). A sensory score of ≤ 6 indicates that the item is unacceptable to be consumed, with a sign of rotten or stinking smell, no shiny color, and overall unacceptability (Alizadeh‐Sani et al. 2017). CH‐TEO wrapped samples and PCH‐TiO_2_ samples had no statistically significant difference in sensory scores, although the CH‐TiO_2_‐TEO wrapped samples had higher sensory attributes scores than those after 12 days of storage because of the antibacterial and antioxidant activities of those. Sensory evaluation of PCH groups was given “unacceptable” scores by the 6th day. On day 9, the PCH‐TiO_2_ and PCH‐TEO films were not acceptable. CH‐TiO_2_‐TEO film best maintained overall acceptability compared to others. In confirmation of this result, similar research was found by Azizi‐Lalabadi et al. (2020) and Alizadeh‐Sani et al. (2017) for lamb and white shrimp, respectively. They found that the application of TiO_2_ nanoparticles effectively improved the sensory attributes. Similarly, Arfat et al. (2015) reported that adding basil leaf essential oil to gelatin‐ZnO nanocomposite could extend the overall acceptability of refrigerated sea bass slices. Sayadi et al. (2022) also declared that the use of TiO_2_ NPs and cumin EO in alginate solution extended the quality of beef. Alizadeh‐Sani et al. (2017) found similar results with using nanocomposite film containing TiO_2_ nanoparticles and rosemary EO to extend the shelf life of lamb meat. Although substantial research suggests that PCH‐TiO_2_‐TEO film could effectively prevent the bacterial growth of fresh fish sensory attributions in the PCH film did not differ significantly in comparison with other groups.

## 4 Conclusion

This study showed that PCH had little effect in killing bacteria or stopping their growth. Analysis of the bacterial results revealed that PCH‐TiO_2_‐TEO groups were excellent nanocomposite films for antimicrobial active packaging of fishery products. TiO_2_ NPs and TEO also displayed good antioxidant properties since POV and TBA values of samples wrapped with these materials were lower than those of the PCH samples over the storage period. The antioxidant activities of PCH‐TiO_2_‐TEO against oxidation of lipids were greater than those of PCH‐TEO films or PCH‐TiO_2_ because of the antagonistic interactions between TiO_2_ NPs and TEO. This research was another confirmation of the biological activities of PCH‐TiO_2_ nanocomposite and PCH‐TEO composite films in the food packaging industry.

## Author Contributions

**Ainaz Khodanazary:** investigation, methodology, conceptualization, writing – review and editing.

## Ethics Statement

The author has nothing to report.

## Conflicts of Interest

The author declares no conflicts of interest.

## Data Availability

The datasets used and/or analyzed during the current study are available from the corresponding author on reasonable request.
